# Arguments against the proposed change of the diagnostic term “idiopathic short stature” into “healthy short stature”

**DOI:** 10.20945/2359-4292-2026-0111

**Published:** 2026-10-05

**Authors:** Jan M Wit, Michael B Ranke

**Affiliations:** 1 Division of Endocrinology, Department of Pediatrics, Willem-Alexander Children’s Hospital, Leiden University Medical Center, Leiden, The Netherlands; 2 Children’s Hospital, University of Tuebingen, Tuebingen, Germany

Dear Editor,

We read with interest the Brief Communication “Healthy Short Stature” by Jorge, Collett-Solberg and Boguszewski in this journal (^1^). The authors present an historical review of the term “idiopathic short stature” (ISS), provide critical comments, and offer an alternative diagnostic label.

Regarding the history, we wish to mention that the choice of the term ISS and its definition, resulting from the first and second (^2^) consensus meetings, ended the confusion due to a wide variety of previous diagnostic labels, and was integrated into Chapter 1 of the ESPE Classification of Paediatric Endocrine Diagnoses (^3^) and its successor [International Classification of Pediatric Endocrine Diagnosis (ICPED), www.icped.org].

A few months ago, a revised version of this chapter was published as Supplementary Information 1 to the International guideline on genetic testing of children with short stature, with Dr. Jorge sharing first authorship with Dr. Dauber (^4^). It proposed that after a full initial clinical evaluation, short children could be first categorized into four classes: isolated short stature (independent of birth size) or three categories of non-isolated short stature (suspicion of skeletal dysplasia, a growth hormone/Insulin-like growth factor-1 axis defect or syndromic short stature). If further diagnostic procedures (including genetic testing if indicated) would reveal the etiology of short stature, the diagnosis can be classified as one of the primary or secondary growth disorders (intrinsic or extrinsic to the growth plate, respectively) (^5^). If not, the provisional diagnostic label “Isolated or non-isolated short stature of unknown etiology with normal or low birth size” can be used, which is further subdivided according to isolated vs non-isolated and normal vs low birth size. Dauber/Jorge and cols. (^4^) thus propose to rename ISS to “idiopathic isolated short stature” (if born with a normal weight and/or length). The term (“isolated”) was added because, as Jorge and cols. mentioned (^1^), “in practice, the term was often applied more loosely to denote short stature of unknown cause, without strict adherence to the recommended diagnostic framework” (^1^).

Jorge and cols. are correct in stating that, since the publication of the ISS consensus statement (^2^), the widespread use of large-scale parallel sequencing (next-generation sequencing, NGS) has shown that in children who would have been labelled ISS in the past, genetic testing can reveal a genetic or epigenetic etiology. According to the Guideline on Genetic Testing (^4^), the logical consequence of detecting a (likely) pathogenic gene variant is that for the respective child the diagnostic label of ISS is changed to the respective primary growth disorder. In agreement with the future perspectives in the guideline (^4^), we speculate that when assessment of the polygenic score for height will become available for clinical practice, “short stature of polygenic origin” may be added to the list of causes of short stature. Similarly, clinical availability of tools reflecting the regulation of gene expression through RNA-sequencing and epigenetic markers will probably reveal new diagnostic labels that will replace a previous label of ISS by a new diagnosis in the respective patient.

In this perspective, it is surprising to read the three critical comments to the use of ISS in the Brief Communication (^1^). The first comment reads: “It is well established that height variation is largely determined by the interplay between numerous common variants with small individual effects and rare variants with moderate or large effects. Thus, in the absence of secondary causes, much of height variation reflects genetic diversity”. The statement is correct, but as long as this cannot be proven in a patient, no other diagnostic label can be used than “idiopathic”.

The second comment reads: “Without broad genetic testing, it is not possible to determine - based solely on clinical findings - whether an individual harbors a pathogenic variant fully or partially explaining their stature”. This is also correct, but does not argue against the use of the diagnostic label of ISS. The term ISS has always been used in the context of the available diagnostic approach. A child who underwent genetic testing five years ago with a negative result can be diagnosed as a primary growth disorder at retesting.

We also agree with the third comment: “Giving the continuous advances in genomics, even currently negative cases may be elucidated in the near future (...). The incorporation of polygenic scores will likely refine our understanding of what is presently labeled as ISS”. However, this is no reason to discard the term ISS. As in other medical areas, the percentage of patients with any diagnostic label starting with the word “idiopathic” will become smaller as medical knowledge and technology advance and expand.

In the following paragraph, Jorge and cols. suggest that “isolated short stature” has been proposed as replacement of ISS. This is not correct. The International Guideline (^4^) has made it clear that this descriptive term can be used after the initial assessment as a start to decide on further testing, and should not be considered a diagnostic category.

Finally, the authors propose to replace the term ISS by “healthy short stature” (HSS), with essentially identical criteria as for ISS. They first suggest that “this terminology more accurately reflects the upper threshold of diagnostic evaluation currently available through comprehensive clinical and laboratory investigation, while also removing the burden of a ‘diagnosis’ for a characteristic that fundamentally represents a normal variant of human growth diversity”. We do not see any indication how this terminology could reflect the “upper threshold of diagnostic evaluation currently available”. Further, the latter part of this sentence suggests that the term HSS reflects certainty that the child’s short stature is caused by normal variation (assumed polygenic origin) instead of the “uncertainty” that is inherent to the term ISS. This apparent “certainty” of the word “healthy” contrasts with the later statement that “the term HSS should not preclude further genetic investigation”.

The authors further suggest that “the concept of HSS emphasizes respect for phenotypic variation and may help to mitigate social and psychological biases associated with stature”. We don’t see how this term could be able to do this, and instead, we believe that parents would not be helped by a statement of the clinician pretending “knowing” that the child is “healthy”, without sufficient evidence. Explaining that current medical technology does not allow for detecting all possible causes of short stature, in combination with the clinicians’ subjective estimate of the likelihood of a polygenic origin without clinical consequences, would serve the patient and parents better.

The authors (^1^) believe that another advantage of using HSS is that “if genetic testing identifies an explanation for the short stature, the designation remains applicable, provided the patient does not manifest additional clinical signs and symptoms”. We do not see why the finding of a genetic abnormality in a short child, whatever the presence or severity of additional clinical features, would not lead to another diagnostic category. This statement is also in strong contrast with Supplementary information 4 (Benefits of establishing the genetic diagnosis in ten prevalent genetic causes detected in children with isolated short stature) to the Guideline on Genetic testing (^4^). It would be odd to keep the term HSS if after obtaining a genetic diagnosis the patient and often also parents are advised to be closely monitored for future complications. And what would payers think about reimbursing clinic visits and diagnostic procedures for “healthy short stature”?

Jorge and cols. anticipate resistance to transitioning from ISS to HSS from the established regulatory and reimbursement frameworks for rhGH therapy and suggest that renaming this condition could generate administrative barriers to treatment access for eligible children. In the few countries where ISS is a registered indication for rhGH therapy this may indeed occur, but we believe that, in the current era of widespread availability of genetic testing, normal results of up-to-date genetic testing should be a mandatory criterion before rhGH can be prescribed for a short child anyhow, either labeled short small for gestational age (SGA) (as recommended in (^4^)) or ISS.

Therefore, we come to opposite conclusions to those of Jorge and cols. (^1^). HSS provides a terminology that is not consistent with current genetic knowledge and uses the word “healthy” without evidence. Using the word “healthy” for a child who is short of unknown etiology is also in contrast to the definition of “Healthy” according to WHO (A state of complete physical, mental and social well-being and not merely the absence of disease or infirmity). If genetic testing at a later age would show a pathogenic gene variant, the doctor will have a hard time to defend his/her previous “diagnosis” of HSS. In the communication with patient and parents, it is better to be honest about uncertainty, and sufficient to present the clinician’s subjective estimate of the likelihood of a (non-)pathologic cause. HSS does not preserve and even less reinforces the need for careful clinical assessment and judicious application of genetic testing when red flags are identified or when new features emerge over time (the parents will argue: “the doctor said that the child is healthy”). By redefining ISS to HSS, one would move away from an inherently provisional but honest idiopathic label and toward a descriptive concept that cannot remain valid alongside future etiological discoveries. This shift would not facilitate the alignment of clinical practice, regulatory frameworks, and research agendas.

## Data Availability

datasets related to this article will be available upon request to the corresponding author.
